# Caseous Necrosis of Mitral Annulus: A Rare Cause of Stroke

**DOI:** 10.1155/2013/748241

**Published:** 2013-05-12

**Authors:** Jérôme Corre, Lionel Leroux, Pierre Coste

**Affiliations:** University Hospital of Bordeaux, Cardiologic Hospital Haut-Lévêque, 33600 Pessac, France

## Abstract

The current report describes a rare case of a caseous necrosis presenting as a pseudotumor in ventricle, revealed by stroke. Cerebral MRI, showing multiples lacunes, evocates a cardioembolic mechanism. Transthoracic and transesophageal echocardiography demonstrate a large hyperechogenic mass fixed to the posterior mitral valve and annulus while thoracic tomography revealed a fully calcified lesion, at the mitral annulus, evocative of caseus necrosis. Medical therapy was preferred (anticoagulation), because of her age and the decaying nature of surgery.

## 1. Case Report

**Figure 1:**
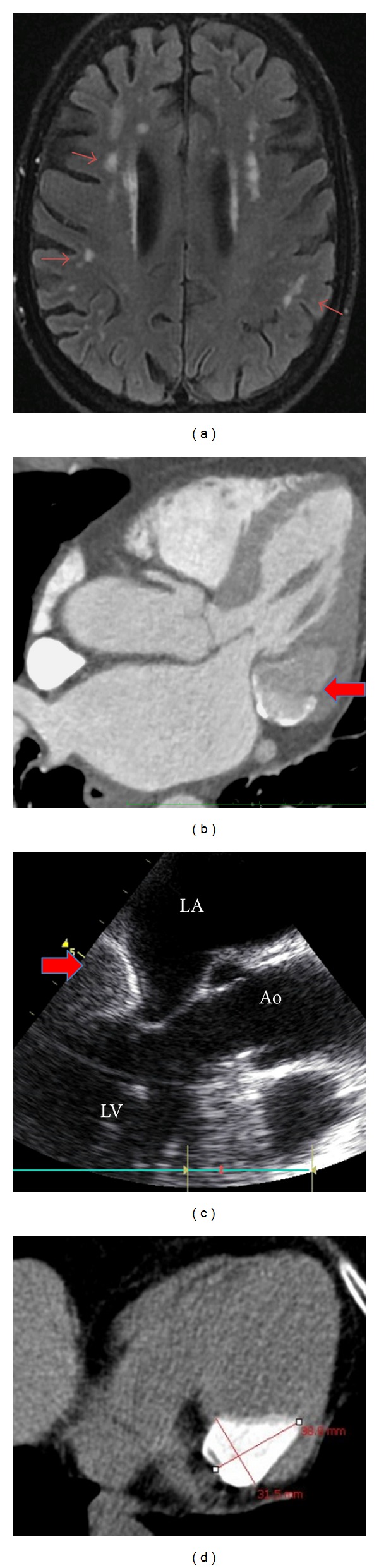
(a) Cerebral MRI shows multiple infarcts. ((b) and (d)) Cardiac CT shows a large calcified tumor at the insertion of the mitral annulus. (c) Transesophageal echocardiography shows a hyperechogenic, unobtrusive tumor fixed to the posterolateral wall of the left ventricle.

An 83-year-old woman, with prior hypertension and dyslipidemia, presented to the emergency room for sudden dysarthria and facial paralysis. Physical examination was unremarkable, except a mitral regurgitation murmur. Biological tests were normal. Cerebral computed tomography (CT) imaging revealed a lacune in the left corona radiata whereas angio-CT showed only moderate atherosclerosis of the carotid arteries. Cerebral magnetic resonance imaging (MRI) (Figure 1(a)) exposed multiple infra- and supratentorial infarcts suggesting cardioembolic strokes.

ECG was normal and 48-hour continuous cardiac monitoring did not reveal arrhythmia. Transthoracic echocardiography (TTE) showed a preserved left ventricular ejection fraction but an increased left atrial size due to a chronic mitral regurgitation (MR). The mechanism of this grade II MR was a posterior mitral leaflet tethering secondary to a large hyperechogenic immobile growth, measured at 39  ×  24 mm, fixed to the posterolateral wall of the left ventricle. There was no mitral stenosis nor aortic valvular disease associated. Cardiac CT (Figures 1(b) and 1(d)) performed in order to analyze this valvular pseudotumor revealed a large calcified tumor, inserted to the mitral annulus evocating caseous necrosis. Transesophageal echocardiography (Figure 1(c)) allowed high-quality images and excluded thrombi and foramen ovale.

We reached a rare case of a caseous necrosis presenting as a left ventricular pseudotumor and revealed by transient ischemic attack. Considering embolic complications, surgical excision was discussed to prevent recurrence, but medical therapy with prolonged anticoagulation was preferred because of difficulties of such a surgery and the advanced age of the patient.

## 2. Discussion

Mitral annular calcification is a chronic, degenerative process of the mitral valve fibrous ring, preferentially affecting the posterior leaflet, and is often associated with risk factors for atherosclerosis [1]. Several studies have demonstrated an association between mitral annular calcification and stroke [2, 3]. The mechanism for stroke is unclear. Thrombus formation on mitral annular calcification with embolization is possible [4], as embolization of small calcified parts of mitral annular calcification [5].

Although there is no consensus on the best therapy, surgery may be preferred, only if the valve can be repaired. Further studies are needed to resolve the optimal treatment to decrease the risk of embolization of mitral annular calcification.
